# *EYS* Mutations Causing Autosomal Recessive Retinitis Pigmentosa: Changes of Retinal Structure and Function with Disease Progression

**DOI:** 10.3390/genes8070178

**Published:** 2017-07-12

**Authors:** David B. McGuigan, Elise Heon, Artur V. Cideciyan, Rinki Ratnapriya, Monica Lu, Alexander Sumaroka, Alejandro J. Roman, Vaishnavi Batmanabane, Alexandra V. Garafalo, Edwin M. Stone, Anand Swaroop, Samuel G. Jacobson

**Affiliations:** 1 Scheie Eye Institute, Department of Ophthalmology, Perelman School of Medicine, University of Pennsylvania, Philadelphia, PA 19104, USA; david.mcguigan@uphs.upenn.edu (D.B.M.); cideciya@mail.med.upenn.edu (A.V.C.); monica.lu@uphs.upenn.edu (M.L.); asumarok@mail.med.upenn.edu (A.S.); aroman@mail.med.upenn.edu (A.J.R.); alexandra.garafalo@uphs.upenn.edu (A.V.G.); 2 Department of Ophthalmology and Vision Sciences, Program of Genetics and Genomic Biology, The Hospital for Sick Children, University of Toronto, Toronto, ON M5G 1X8, Canada; elise.heon@sickkids.ca (E.H.); vaishnavi.batmanabane@sickkids.ca (V.B.); 3 Neurobiology Neurodegeneration and Repair Laboratory, National Eye Institute, National Institutes of Health, Bethesda, MD 20892, USA; rinki.ratnapriya@nih.gov (R.R.); swaroopa@nei.nih.gov (A.S.); 4 Department of Ophthalmology and Visual Science, Stephen A. Wynn Institute for Vision Research, Carver College of Medicine, University of Iowa, Iowa City, IA 52242, USA; stonee@zeus.eng.uiowa.edu

**Keywords:** optical coherence tomography, rod, cone, autofluorescence, ciliopathy

## Abstract

Mutations in the *EYS* (eyes shut homolog) gene are a common cause of autosomal recessive (ar) retinitis pigmentosa (RP). Without a mammalian model of human *EYS* disease, there is limited understanding of details of disease expression and rates of progression of the retinal degeneration. We studied clinically and with chromatic static perimetry, spectral-domain optical coherence tomography (OCT), and *en face* autofluoresence imaging, a cohort of 15 patients (ages 12–51 at first visit), some of whom had longitudinal data of function and structure. Rod sensitivity was able to be measured by chromatic perimetry in most patients at their earliest visits and some patients retained patchy rod function into the fifth decade of life. As expected from RP, cone sensitivity persisted after rod function was no longer measurable. The photoreceptor nuclear layer of the central retina was abnormal except at the fovea in most patients at first visit. Perifoveal disease measured over a period of years indicated that photoreceptor structural loss was followed by dysmorphology of the inner retina and loss of retinal pigment epithelial integrity. Although there could be variability in severity, preliminary analyses of the rates of vision loss suggested that *EYS* is a more rapidly progressive disease than other ciliopathies causing arRP, such as *USH2A* and *MAK*.

## 1. Introduction

Understanding of disease expression in inherited retinal degenerations (IRDs) has benefited from in vitro studies, animal models that faithfully mimic the human condition, and histopathology of post-mortem retina donors. Mutations in the *EYS* (eyes shut homolog) gene, originally designated as the RP25 locus on chromosome 6q12, are a major cause of autosomal recessive (ar) retinitis pigmentosa (RP) [1,2,3,4,5]. There are no mammalian models of human *EYS* disease [6,7], but recently there was a report of retinal histopathology of three post-mortem donors with IRD due to *EYS* mutations [8]. The donors were elderly (ages 72, 91 and 97) at the time of death, but there were some notable findings. There were no cones in the peripheral retina; rhodopsin labeled cells were rare or absent in the analyzed areas (periphery and perifoveal regions); the perifovea was described as showing a prominent inner nuclear layer (INL) with patchy disorganized cones; and there were localized areas of retinal pigment epithelium (RPE) atrophy in the perifovea.

Prompted by these clues about later-stage *EYS* disease, we studied a cohort of *EYS*-RP patients with non-invasive measures of photoreceptor function and structure and of RPE integrity. We sought to determine the disease progression that could be leading to these later stage findings shown by the histopathology. Many of our *EYS*-RP patients were able to be studied longitudinally and this provided a glimpse of the natural history of this relatively common form of arRP.

## 2. Materials and Methods

### 2.1. Subjects

Fifteen unrelated patients with retinal degeneration caused by *EYS* mutations were included in this study (Table 1 and Table 2). This retrospective study involved two clinical sites: the Scheie Eye Institute in Philadelphia and the Hospital for Sick Children in Toronto. Informed consent was obtained and all procedures were approved by our institutional ethics review boards and adhered to the Declaration of Helsinki. The ethical approval numbers of the studies are 226,100 and 704,353. Patients underwent a complete eye examination and ancillary tests. Electroretinography (ERG) was not a focus of this work, but 14 patients did have ERGs following an International Society for Clinical Electrophysiology of Vision (ISCEV) protocol.

### 2.2. Genetic Analysis

Commercially available molecular diagnostic tests performed at Clinical Laboratory Improvement Amendment (CLIA) approved laboratories were used for analysis of most samples in this cohort. Samples were sequenced using next generation sequencing (NGS), and mutations were confirmed with Sanger sequencing (P1–P4, P6–12 and P14). P5 was tested using Sanger sequencing in a CLIA-approved laboratory. As part of clinical diagnostic testing, comparative genomic hybridization (CGH) array analysis of P3 and P7 was used for detection of gross deletions in these patients. The deletion of exon 12 in P10 was confirmed by repeating the PCR amplification using multiple primer sets, followed by NGS and then Sanger sequencing in a CLIA-approved laboratory. Whole exome sequencing was used to detect mutations in P9, P13 and P15.

### 2.3. Psychophysics

Goldmann kinetic perimetry was performed with V-4e and I-4e test targets. Field extent was quantified with a previously described computer-based algorithm [9,10]. The field extent in steradians was converted to a percentage of the mean normal field extent [10]. The kinetics of field extent loss was determined for a subset of patients according to a model of exponential decay [11]. The annual rate of field loss for each individual was calculated by taking a linear regression through log-transformed field extents; the slope of the regression is the rate of loss. Four patients were included for this analysis and two patients were excluded: P7 did not have sufficient follow-up time for kinetic perimetry (0.9 years) and P9 had an excessively long half-life (42 years) which would skew the analysis. The latter patient at first visit was already reduced to a central island of vision and this remained a central island of similar extent for the next 14 years.

Static perimetry was performed with a modified perimeter (Humphrey Field Analyzer 750i) as previously described [12,13]. Light-adapted and two-color dark-adapted function was measured at 2° intervals across the central visual field (central 60° along horizontal and vertical meridians) and at 12° intervals throughout the visual field. Photoreceptor mediation under dark-adapted conditions was determined by the sensitivity difference between the 500- and 650-nm stimuli [12,13].

### 2.4. Imaging

Optical coherence tomography (OCT) was used to analyze laminar architecture across the central retina. Retinal cross-sections were recorded mainly with a spectral-domain (SD) OCT system (RTVue-100; Optovue Inc., Fremont, CA, USA) and some with time-domain (TD) OCT instruments (OCT1, OCT3; Carl Zeiss Meditec, Dublin, CA, USA). Post-acquisition data analysis was performed with custom programs (MatLab 7.5; MathWorks, Natick, MA, USA). Our recording and analysis techniques have been previously described [14,15,16,17,18]. Longitudinal reflectivity profiles (LRPs) were used to identify retinal features. The hyposcattering outer nuclear layer (ONL) was defined between the hyperscattering outer plexiform layer (OPL) and the hyperscattering outer limiting membrane (OLM). Some retinal regions lacked the signal peak corresponding to the OPL which separated the ONL from the other major hyposcattering layer, the inner nuclear layer (INL). The resulting contiguous hyposcattering layer was termed INL+. The width of the inner segment/outer segment (IS/OS) band was measured from nasal to temporal end points according to previous methods [19,20,21]. A rate of IS/OS width constriction was calculated for patients with serial data; P6 with cystoid macular edema was excluded. A model of exponential decay was used [22]. For comparison with a previously reported cohort of *EYS* patients, the rate of constriction was calculated using an alternative model of linear progression [23].

A confocal scanning laser ophthalmoscope (Spectralis HRA; Heidelberg Engineering, Heidelberg, Germany) was used to record *en face* images and estimate RPE health. Near-infrared reduced-illuminance autofluorescence imaging (NIR-RAFI) and short-wavelength reduced-illuminance autofluorescence imaging (SW-RAFI) were performed using methods as previously described [24,25]. For NIR-RAFI, excitation at 790 nm was used at 100% laser power setting and 105% detector sensitivity. For SW-RAFI, excitation at 488 nm was used at 25% laser power and 105% detector sensitivity. All images were acquired with high speed mode (30° × 30° square field sampled onto 768 × 768 pixels) and with the automatic normalization feature turned off. The manufacturer’s ART feature was used whenever possible with a 21-frame average.

## 3. Results

### 3.1. Clinical Characteristics of the EYS Patients in this Study

Fifteen patients were identified with two mutations in the *EYS* gene (Table 1). Missense mutations, truncating mutations, gross deletions and splice-site mutations were represented in this cohort, reflecting the diversity of mutations known to occur in this gene. In total, eight of the 21 distinct mutations found in this cohort were previously not reported. Of note, patients P3, P7 and P10 exhibited gross deletions in at least one allele of the *EYS* gene. P3 had a novel deletion of exons 15-18, whereas P7, with a deletion of exons 15-19, and P10, with a deletion of exon 12, had mutations similar to those previously described [1]. Patients P2, P4 and P11 had mutations that are predicted to affect splicing. Ages at first visit ranged from 12 to 51 years (Table 2). Fundus appearance in all of the patients was that of extramacular retinal degeneration including attenuation of retinal vessels, pigmentary retinopathy (bone spicule-like pigment and rarely, small clumps of pigment), a waxy pale optic nerve head, and, at later ages in some patients, some chorioretinal atrophy. Macular fundus appearance was less affected by pigmentary abnormalities except in P14 who had atrophic lesions in the central retina. ERG data were available in 14 patients at or near the age of first visit. Considering the rod and cone ERG waveforms, six patients (P6, P7, P9, P10, P13, P14; age range, 24–47 years) had no detectable ERG to rod or cone stimuli. Among the remaining eight patients, rod ERG b-waves were either severely reduced in amplitude (P8, age 32) or not detectable (P1–P5, P12, P15; age range, 10–50 years); cone ERGs were reduced in amplitude. In summary, the ERGs, when measurable, were those of patients with rod>cone dysfunction.

Visual acuities were at least 20/30 in the first decades of life (Table 2); our cross-sectional data indicated a decline with age. An estimate of rate of visual acuity decline in *EYS* patients was calculated from serial measurements in the eight patients with follow-up times of >3.5 years. The annual exponential rate was 5.7% per year (0.024 logMAR/year; logMAR: logarithm of the Minimum Angle of Resolution).

Kinetic visual fields were measured in 11 patients and serial data (with an interval of >3 years) were available for five patients. Fields from the five patients at different ages are shown (Supplementary Materials Figure S1). P3 at age 19 had a full field (large target) with absolute scotomas in the midperiphery and near (but not encroaching on) fixation. By age 21, the more pericentral scotomas coalesced and surrounded the small central island of function. A complete annular midperipheral and pericentral scotoma was evident at age 23; by age 27, there was only a small central island and a temporal peripheral island. The small central island remained at age 33. P4 showed a relatively similar pattern of visual loss as P3 with midperipheral absolute scotomas at ages 19 and 21 progressing to only residual central and peripheral islands by age 37 and then only a central island by age 42. The dimension of the retained central island at all ages in P4 was larger than in P3. P5 showed erosion of vision in the midperiphery at age 24 but the retained island at age 27 demonstrated a loss of far peripheral function and more inferior preservation of field. Eventually, at age 39, P5 had only a residual central island of function. P9 at age 34 was already reduced to a central island of field and scattered small islands temporal to it. At ages 40 and 48, only the central island remained. This is in contrast to P15 at age 51 who had a relatively extensive field remaining; over the next few years, however, the midperipheral scotoma isolated the central island from the peripheral temporal island, as in P3 (age 23) and P4 (age 25).

Visual field extents (V-4e target) of the 11 patients were plotted as a function of age (Supplementary Materials Figure S1G). The kinetics of visual field loss according to a model of exponential decay were studied in a subset of these patients (*n* = 4; follow-up time: 3–27 years). The average rate of field loss was 23.1% per year.

### 3.2. Cone and Rod Function Determined Perimetrically in EYS

#### 3.2.1. Peripheral Cones and Rods

**Figure 1:**
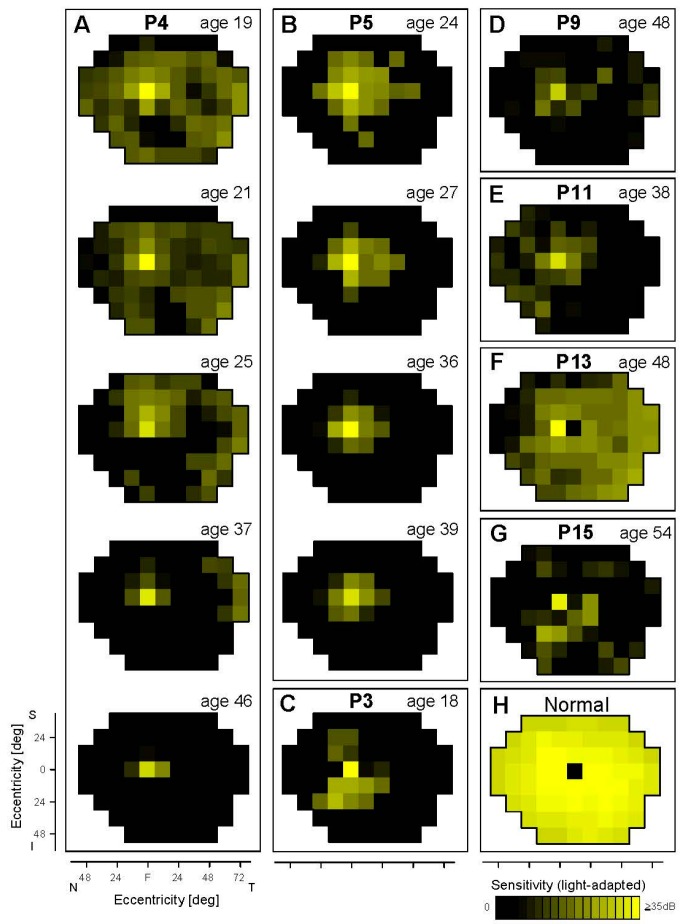
Cone sensitivity across the visual field in *EYS* patients. Light-adapted perimetry results of seven patients are shown as maps (**A**–**G**). P4 (**A**) and P5 (**B**) have serial maps from visits spanning decades. Normal cone sensitivity is shown for reference (**H**). The physiological blind spot is represented as a black square at 12° in the temporal field. N, nasal; T, temporal; S, superior; I, inferior visual field.

There were no detectable cones in the periphery of the donor retinas [8]. Peripheral cone sensitivity, however, was detectable albeit abnormally reduced in five of the seven *EYS* patients studied with cone perimetry; the age range was wide (oldest being P15 at age 54; Figure 1). P4 at age 19 had measurable cone sensitivity in the peripheral field, although reduced by ~20 dB. Peripheral cone sensitivity progressively diminished until age 37 when there was only a temporal island of reduced function; at age 46, there was no measurable peripheral cone function. P5 had a more limited amount of extracentral cone sensitivity at first visit (age 24) and these loci, on average, had 15 dB of cone sensitivity loss. At age 27 and through age 39, there was only residual central cone function with progressively diminishing sensitivity. P3, at age 18, already had no measurable peripheral cone function; P9 at age 48 retained a central island but also limited temporal peripheral islands of barely detectable cone function. Of interest, there were regional differences in the peripheral cone function: P11 (age 38) had residual nasal field function; P13 (age 48) had considerable temporal field cone function; and P15 (age 54) had remaining inferior field cone function. These in vivo results indicated that peripheral cone function (implying persistence of cones) could be present at a variety of ages, but was severely abnormal even at the earliest ages we studied. Whether sampling of the nasal retina in the eye donors would have revealed residual cones corresponding to their temporal visual fields is unknown.

Immunostaining in the periphery of two donor retinas showed some rhodopsin-labeled cells, but this was only rarely noted in the other donor retina; rhodopsin was also detected outside the outer segments. Rods had abnormal morphology [8]. P3 at 18 years, the earliest age studied, showed a few detectable loci in the inferior midperipheral field with rod-mediated function and these had 20 dB of rod sensitivity loss. P4, P5, P9, P11, and P13, representing earliest ages examined of 19, 24, 34, 38 and 48 years, had little or no detectable extracentral rod function. P15 at age 54, however, retained minimal peripheral rod function in the inferior field, but there was 20–30 dB of rod sensitivity loss. These results, taken together, indicate that there is major peripheral rod cell dysfunction and probable degeneration by the second decade of life; persistence of abnormal peripheral cone function, however, could be detected into the fourth, fifth and sixth decades of life (Figure 1).

#### 3.2.2. Central Cones and Rods

**Figure 2:**
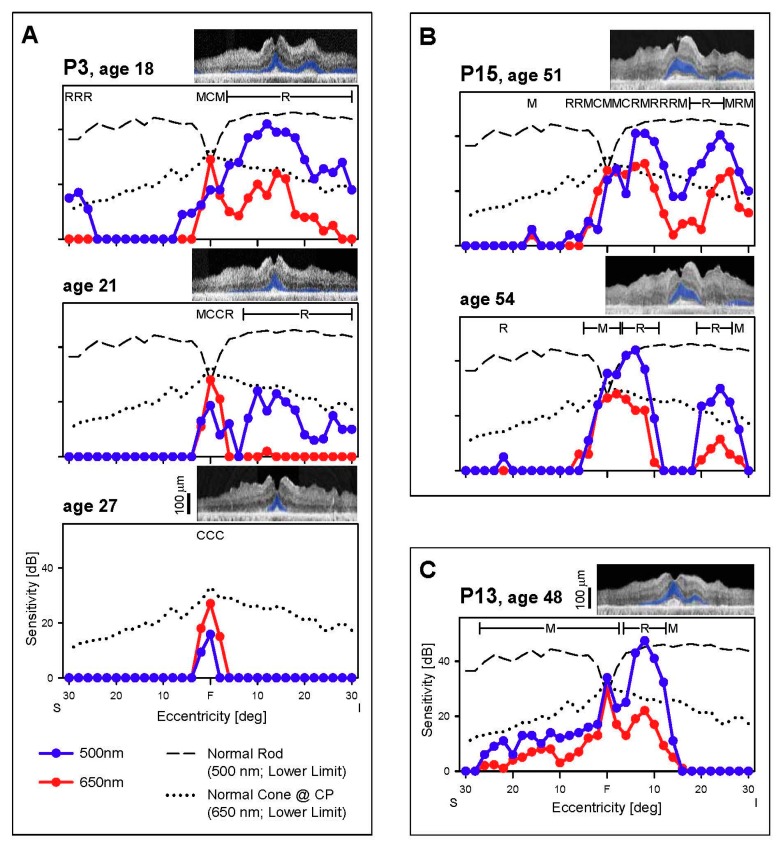
Rod and cone function across the vertical meridian. (**A**–**C**) Dark-adapted, two-color profiles measured across the central 60° in 3 *EYS* patients. Photoreceptor mediation of the 500 nm (blue symbols) and 650 nm (red symbols) stimuli is based on the sensitivity difference between the two colors and is shown above the data: R, rod; M, mixed rod and cone; C, cone-mediated. Interrupted lines are the lower limit of normal sensitivity (-2SD) for rods to the 500 nm stimuli (long dashes) and for cones to the 650 nm stimuli (dots). All three patients present with abnormal rod and cone function and this further diminishes, as demonstrated in the two patients with serial data (P3, P15). Insets, Optical coherence tomography (OCT) images colocalized with perimetry; outer nuclear layer (ONL) colorized blue for visibility. F, fovea; S, superior and I, inferior visual field.

**Figure 3:**
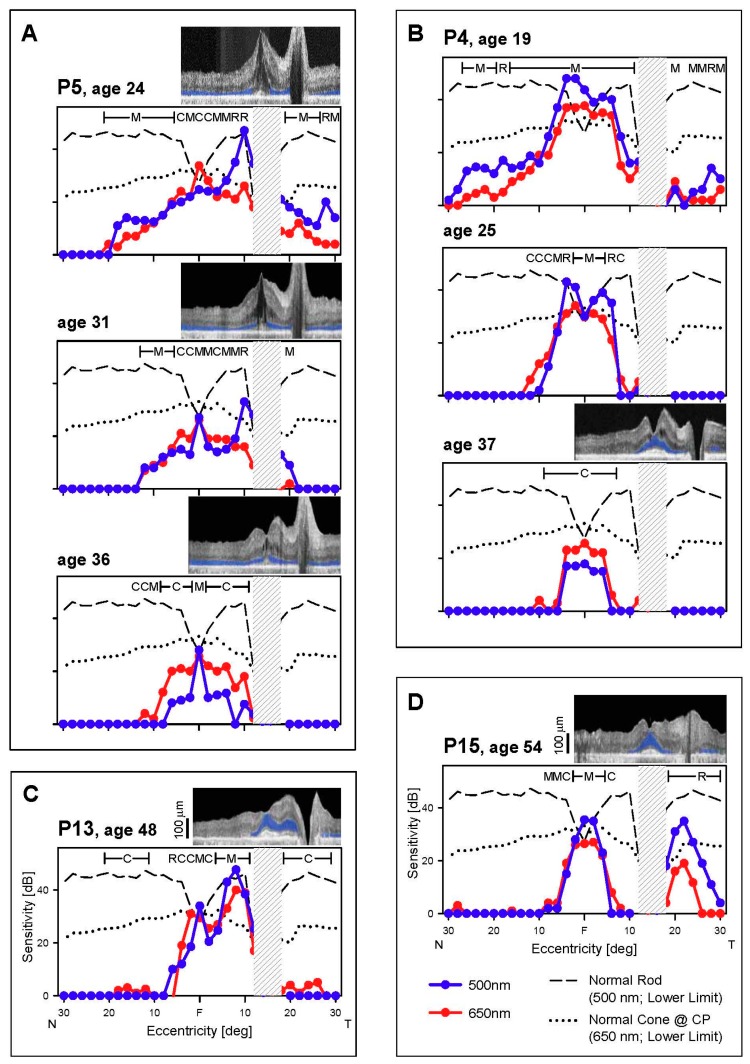
Rod and cone function across the horizontal meridian. (**A**–**D**) Dark-adapted, two-color profiles measured across the central 60° in four *EYS* patients. All patients present with reduced rod and cone function. P5 (**A**) and P4 (**B**), the two patients with serial data, show progression of dysfunction to central islands of mainly cone function at the later visits. Hatched bar, location of physiological blindspot. Normal lower limits depicted as in Figure 2. Insets, OCTs colocalized with perimetry. F, fovea; N, nasal and T, temporal visual field.

No rhodopsin-labeled cells were present in two donor eyes and only some disorganized rod cells were noted in the other donor. A few perifoveal cones remained but had abnormal opsin localization by immunocytochemistry [8]. We examined our in vivo data to try to understand what may have preceded the end stage degeneration in the donor retinas. There was substantial rod and cone function in the central field at the earliest stages in five of seven patients (Figure 2 and Figure 3). The location of these patches of rod and cone function could be inferior (in the field) to the fovea (P3, P13, P15; Figure 2A–C) or adjacent to the optic nerve on either the temporal field side (P15; Figure 3D) or nasal field side (P5, P13, Figure 3A,C), or in a broad central island (P4; Figure 3B). Cross-sectional OCT images at the same visit of the patients showed detectable ONL that generally corresponded to the photoreceptor function. In those patients with serial data, rod function diminished over time, leaving mainly a cone central island at late stages. As exemplified by the dark-adapted 650 nm sensitivities in the central few degrees (Figure 2 and Figure 3), residual foveal cone function could be within or near normal limits, consistent with the normal or near normal visual acuities in these patients (Table 2).

### 3.3. Retinal Laminar Architecture by OCT

**Figure 4:**
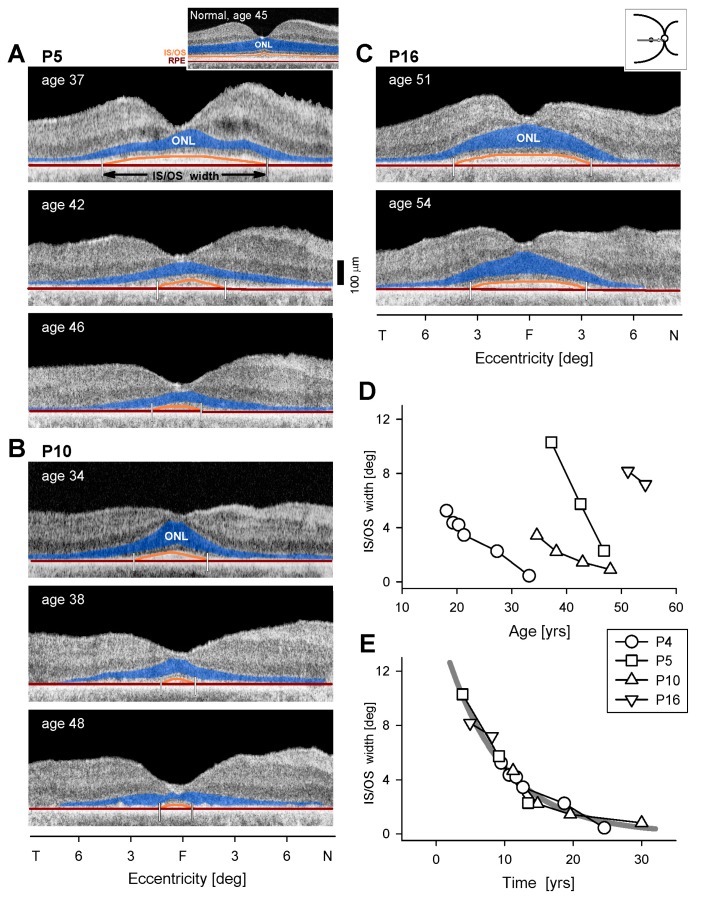
Serial changes in central retinal structure in *EYS*. (**A**–**C**) OCT scans along the horizontal meridian through the fovea in three *EYS* patients. ONL is colorized in blue and proximal edges of the inner segment/outer segment (IS/OS) band and retinal pigment epithelium (RPE) used for segmentation are colored in orange and dark red, respectively. Small vertical lines mark endpoints of IS/OS width. Inset above (**A**) shows a normal central retina, also with colorizing of ONL, IS/OS and RPE. (**D**) IS/OS width plotted as a function of age for four patients; serial data connected by lines. Note: symbols in D and E are the same and identified in the key between the two graphs. (**E**) Longitudinal measurements of IS/OS width from each patient are shifted along the time axis to fit the model of exponential decay; the rate is 12% per year (thick gray line).

All seven patients with OCT data (P3, P4, P5, P9, P11, P13, P15) retained a central island of ONL which could be subtly asymmetric (Figure 4), corresponding to the asymmetric visual function described earlier (Figure 2 and Figure 3). There was greater ONL preservation in the superior than inferior retina in P3, P13, and P15 and also more ONL preserved in the nasal than temporal retina in P9, P13, and P15. At the earliest visits with available imaging, foveal ONL thickness was normal in only three patients: P3 (age 18), P13 (age 48), and P15 (age 51; Figure 4C). The IS/OS band was detectable, although abnormally reduced in extent, in all seven patients. This hyperreflective band, also known as the ellipsoid zone (EZ) line, corresponds to a region near the junction of the inner and outer segments of rods and cones and is easily discernible across a normal retina [33,34]. The width of the IS/OS band over time was used to evaluate disease progression in a subset of 4 patients with serial data (average follow-up time, 10.3 years; average interval between visits, 3.7 years). P3 (OCT not shown), P4, P9, and P15 had an IS/OS band restricted to the central 4.6° to 10.3° at the earliest visit (IS/OS traced in orange, Figure 4A–C). IS/OS width was constricted and was barely detectable at later visits for P3, P4, and P9. This progression was plotted (Figure 4D). Two models of progression were considered. To permit comparison with a previously reported cohort of *EYS* patients [23], a linear model showed a constriction rate of 5.8% per year when the initial IS/OS extent of each patient was normalized to 100%. An exponential decay model [35] showed a constriction rate of 12% per year. Data from all patients appeared to be well fit qualitatively by an exponential decay function when shifted along the time axis (Figure 4E), consistent with exponential progression models previously used in retinal degenerations [22,36].

The perifoveal sections in the published histopathology of the *EYS* donor retinas were described as having a “prominent inner nuclear layer” [8] (p. 295) as shown in Figure 4 therein. The retinas appeared generally thinned and lamination was abnormal. Compared to the normal control perifoveal histology shown, the section from Donor 1 had a defined INL, an apparently thinned OPL and an ONL that may represent disorganized cones. The RPE was described as thinned. Donors 2 and 3 showed greater retinal disorganization than Donor 1. The boundary between INL and outer retinal layers was less discrete and there may have been not only cone remnants but also glial elements. An RPE layer was present in part of the section from Donor 2, but not clearly discernible in Donor 3. We asked whether there was a sequence of abnormalities that could lead from early disease to these examples of late-stage retinal degeneration.

**Figure 5:**
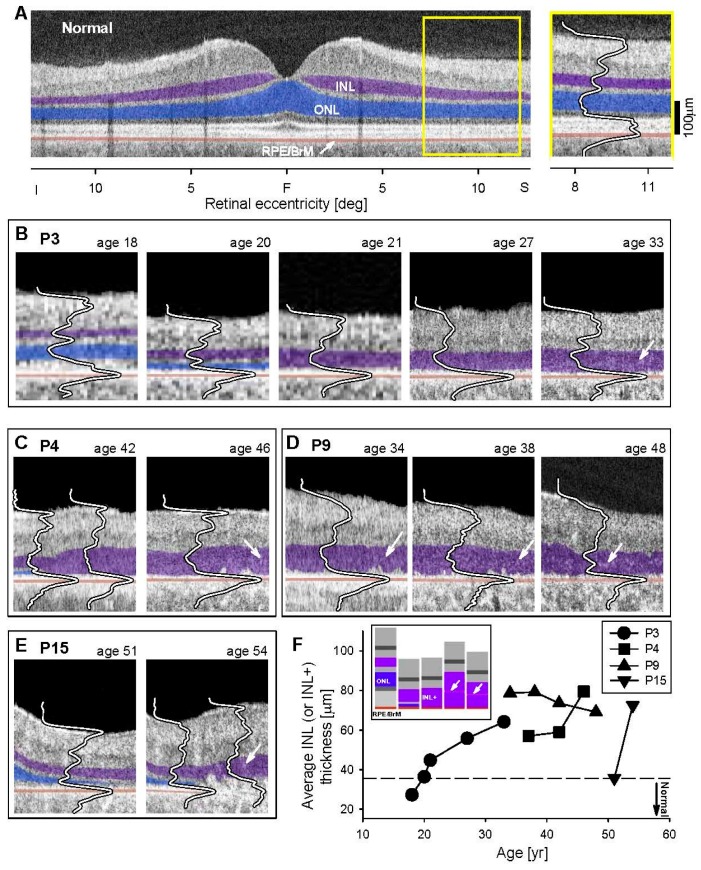
Progression of inner retinal structural abnormalities in *EYS* patients. (**A**) Cross-sectional OCT along the vertical meridian through the fovea in a representative normal retina with inner nuclear layer (INL), outer nuclear layer (ONL), and RPE/BrM (retinal pigment epithelium/Bruch’s Membrane) colorized for visibility with purple, blue, and orange, respectively. Enlarged view of a 5° wide perifoveal region in the superior retina (marked as a yellow rectangle on the unmagnified scan) with an overlaid longitudinal reflectivity profile (LRP). (**B**–**E**) Corresponding sections in four *EYS* patients sampled serially. A thinned ONL layer was sometimes present at early visits (P3, P4, P15) but was not detectable on later visits which showed abnormally thickened INL (termed INL+). White arrows point to hyperreflectivity within the INL+. (**F**) Average INL (or INL+) thickness is plotted as a function of age for four patients; serial data are connected by lines. Dashed line represents upper limit (+2SD) of normal INL thickness. Inset (upper left corner of graph) is a schematic of the postulated changes in laminar architecture over the disease course. F, fovea; S, superior; I, inferior retina. INL+, designation for abnormal inner retinal layer noted when ONL is no longer discernible.

A normal vertical OCT section across the fovea is shown, highlighting ONL, INL and RPE/Bruch’s Membrane (Figure 5A); there is an adjacent magnified section superior to the fovea. A sequence of five OCTs from P3 (at the same superior retinal region as the normal) representing a 15–year timespan from ages 18 to 33 suggests there may be an understandable progression of disease (Figure 5B). At age 18, the ONL and INL (and retinal thickness) were within normal limits. This relates to the relatively normal patches of rod and cone function previously shown (Figure 2A). At age 20, retinal and ONL thickness had decreased and INL was apparently increased. Such observations of thickening INL coupled with thinning ONL have been noted in other IRDs [15,37,38] and there has been support for this OCT observation in retinal histopathology of murine models of retinopathies [38,39]. Between ages 21 and 33, the OPL was no longer discernible resulting in a single, thick, hyporeflective layer which we term INL+. In P4, over an interval of four years (ages 42–46), there was initially a small ONL layer at one edge of the scan and this was replaced by an abnormally thick INL+ layer (Figure 5C). As mentioned in the histopathology of *EYS*, the hyperreflective foci adjacent to the RPE may have been abnormal RPE cells migrating into the outer retina (age 46). P9 had no obvious ONL in the section and only a thickened layer, presumably comprised of INL, hypertrophied glial cells, or even remnant photoreceptors (Figure 5D). A subtle hyperreflective band developed within this INL+ layer at latest visits in P3, P4 and P9. P15, at ages 51 and 54 years, had detectable ONL which became thinner and then not discernible, while INL became thicker. Loss of RPE integrity was notable at age 54 in the superior half of the section and there may have been migration of these cells into the retina.

Serial OCT data from the patients are plotted and show the progressive thickening of the INL or INL+ (Figure 5F). In P9, there is also a downturn of layer thickness. A schematic of the possible changes in layers over the disease course is shown (Figure 5F, inset).

### 3.4. RPE Disease in EYS

**Figure 6:**
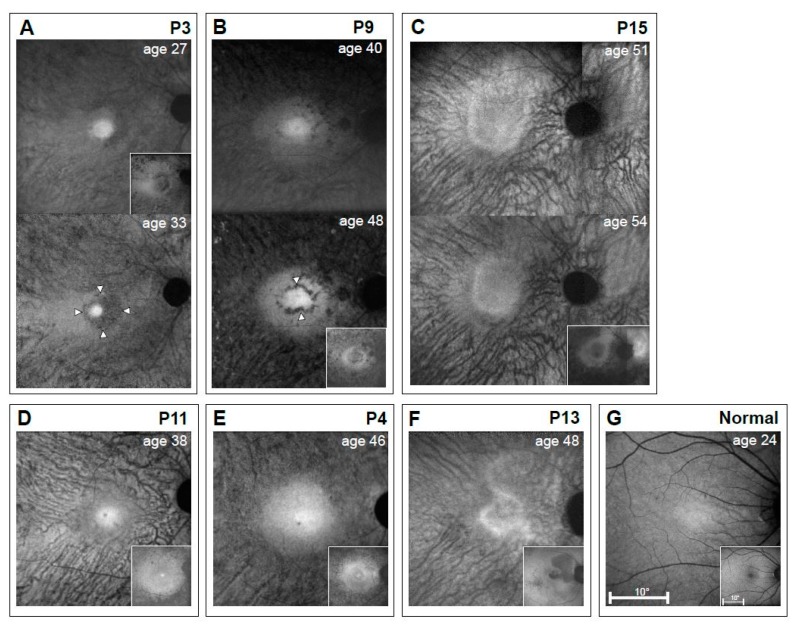
Autofluorescence imaging results of the macula of a representative normal subject compared with 6 *EYS* patients. Melanin autofluorescence with Near-infrared reduced-illuminance autofluorescence imaging (NIR-RAFI) is shown in the main panels, and lipofuscin autofluorescence (when available) with short wavelength (SW)-RAFI is shown as insets (lower right panels). (**A**–**C**) Serial data from P3, P9, and P15 are over six, eight, and three years, respectively. (**D**–**F**) P11, P4, and P13 have images at single timepoints. (**G**) Images from a 24-year-old normal subject. All eyes are displayed as equivalent right eyes and images are individually contrast stretched for visibility of features. Arrowheads in P3 (age 33) and P9 (age 48) indicate hypo AF rings around the hyper AF central signal.

Histopathology revealed focal loss of RPE in the perifovea of two donor retinas and uniformly thinned RPE in the other [8]. NIR-RAFI is an in vivo method to estimate RPE melanin integrity; in normal subjects, there is a circular central region of higher brightness and surrounding lower brightness, which is relatively homogeneous across the fundus [24]. SW-RAFI arises from RPE lipofuscin; normal SW-RAFI shows a region of reduced signal at the macula, due to the absorption of SW light by macular pigment, and there is higher signal surrounding it (Figure 6G). The combination of NIR-RAFI and SW-RAFI analysis allows for delineation of likely regions of RPE atrophy [25].

Seven *EYS* patients with NIR-RAFI showed perifoveal RPE abnormalities and more central regions of increased signal that suggested better RPE preservation. Similar to many other forms of inherited retinal degeneration, the higher AF signal was circular or slightly elliptical [37,39,40]. P3, P4, P9 and P11 at the earliest ages recorded (Figure 6) showed the central circle of higher AF with or without a surrounding annulus of lesser brightness and then a choroidal signal representing a greater loss of RPE melanin. After a six-year interval in P3 (age 27–33), the central AF diminished in diameter and a ring of lower signal was evident (Figure 6A, lower panel). P9, over an eight-year interval (age 40–48), showed a more prominent but still incomplete dark ring around the central high AF (Figure 6B). In general, the extent of the higher central AF in all patients corresponded to retained photoreceptor function (by perimetry) and structure (by OCT). P3, P4, and P9 show regions of reduced AF signal in the SW-RAFI images (insets) that match the perimacular areas of demelanized RPE and choroidal visibility by NIR-RAFI implying RPE atrophy. P11 demonstrated a larger region of retained lipofuscin signal that extended beyond the region of central hyper AF on NIR-RAFI (Figure 6D) implying retained yet demelanized RPE cells across the macula.

P13 and P15 (Figure 6C,F) had a different pattern of NIR-AF than the other patients shown. The central area of higher AF was not circular but polygonal. Surrounding the center was the choroidal-like signal as in the others. In P13, the relatively hypo AF anisotropic area in the SW-RAFI image represents relatively healthy RPE, while hyper AF across the rest of the macula and perimacular area is interpreted as retained but demelanized RPE. P15 illustrates a non-circular pattern with both NIR-RAFI and SW-RAFI; there was a region of higher AF signal on the nasal retinal side of the optic nerve head at both ages and this corresponded to the rod and cone function and ONL in that region (Figure 3D).

## 4. Discussion

### 4.1. Comparing EYS-RP Progression to That in Other Ciliopathies

How does *EYS*-RP compare with other ciliopathies causing arRP in terms of a time course of progression? Very few serial data of vision have been reported for *EYS*-RP [41]. Our limited sample of longitudinal data of kinetic fields (*n* = 4) suggests a relatively rapid time course of peripheral visual field loss for the V-4e test target of about 23% per year. *MAK*-RP (male germ cell-associated kinase), on the other hand, is considered to be a mild disease [42,43] and field loss in these patients indicated a result of 11% per year for the V-4e target (*n* = 60) [42]. *USH2A* has had quantitation of kinetic fields and based on data from nine patients, there was also an ~11% per year decline [37]. From the data at hand in these three ciliopathies, it can be suggested that *EYS* peripheral disease progression is faster than that of the *MAK* and *USH2A* phenotypes. If such results are confirmed in larger series, then any therapeutic attempts to slow the natural history of *EYS*-RP could determine efficacy in a shorter time span than in most other forms of RP. Visual acuities from the *EYS*-RP patients were analyzed and compared with those of the two other arRP ciliopathies. There were significant differences among the three diseases in terms of the longevity of visual acuity. For the event of visual acuity crossing a criterion of 20/32, the *EYS* group showed a median age of 36, compared to 48 and 69 years for *USH2A* and *MAK*, respectively. Survival curves were statistically different across groups (*p* < 0.05, log-rank test; each group had *n* = 8 patients). In summary, visual acuity in *USH2A* and *MAK* was better preserved than in *EYS* by one and three decades, respectively. Such data do not substitute for a natural history study of *EYS*-RP patients in anticipation of a clinical trial of therapy, but do provide an opportunity to advise molecularly-characterized patients about prognosis. 

Are there any objective measures of change in *EYS* patients? IS/OS border width measured across OCT central retinal scans has been proposed as a sensitive and reliable measure of disease progression [19,44]. In a subset of our *EYS* patients with serial OCT data (*n* = 4), we calculated a rate for IS/OS shortening of 13 ± 8% per year, assuming a model of exponential decay [22,35]. IS/OS width was recently examined in a larger cohort (*n* = 12) of *EYS* patients [23]. Under an assumption of linear progression, the rate of shortening of IS/OS width (across horizontal OCT scans) relative to baseline width was 5.2 ± 3.1% per year for an average initial IS/OS width of 3.1 mm (10.3°). We recalculated our rate according to this model and found good agreement: 5.6 ± 2.6% per year for an average initial IS/OS width of 2.0 mm (6.5°).

### 4.2. Inner Retinal Abnormalities in EYS

The histopathology in retina donors with *EYS*-RP reported a prominent INL in the perifoveal sections [8]. This observation led us to focus on this same retinal region in vivo by OCT and, for the first time, we were able to examine serial data over more than a decade in a rapidly progressive disease and document the transformation from normal laminar architecture to the retinal disorganization we previously noted in many IRDs [15,37,38,39,45,46,47,48,49]. The sequence of changes from these in vivo data are likely those that correspond to the complex process of retinal remodeling, as has been described in animal models and human post-mortem retinal tissue from patients with IRD [50,51,52]. A detailed explanation for the increase in INL (or INL+) thickness (after ONL loss) has not been provided; whereas this is only one of many exceedingly complicated and continuous retinal changes after photoreceptor loss, it could be a marker for a stage of remodeling that would be useful to understand in the planning of clinical trials. In a limited way, we captured such abnormalities by histology in certain animal models [38,39] and confirmed the merging of remnant ONL and INL and increased INL layer thickness. More detailed studies, such as are able to be performed using various histopathological and immunocytochemical techniques in the animal models, are needed to explore the basis of this relatively common OCT observation in degenerating retina [53,54].

The OCT images in the *EYS*-RP patients show a resemblance to the images from the pericentral retina in the eye donor histopathology. The mingling of ONL and INL layers shown in the histopathology occurs as OPL is thinned and synaptic contacts diminish. The thickened INL is thus, as we previously speculated, a layer that is more a mixture of cell layers and probably glial elements. For the first time, we have also shown that this INL+ layer can eventually thin. Whether this is due to glial cell retraction or reduction in neurons is not known. The notion of optogenetic therapies being directed at the INL, and specifically bipolar cells, may require further study of the remodeling process in the animal models—with a specific attempt to relate the findings in the degenerating retinas to the in vivo findings in human disease [50,52]. Also noted in these dysmorphic retinas was a discontinuous hyper-reflective band within the INL+ layer. For lack of greater understanding of its morphological basis, we can describe this as a pseudo-OPL. Among many possibilities, these could be new neurites from all cell types appearing in the INL, forming patchy foci in the remnant OPL [51,52].

### 4.3. Mutation Spectrum in this EYS Cohort Versus Others

How does the mutation spectrum observed in our cohort compare to that of the mutational landscape of *EYS*? The mutations that we observed in our cohort are mapped to the EYS protein along with 197 previously reported pathogenic or likely pathogenic mutations, which represent the majority of known *EYS* mutations (Supplementary Materials Figure S2). Mutations were classified as either truncating, missense, gross deletions or splice-site mutations. Mutations that affect 5′ UTR mutations were not included in the diagram or analysis, and none of the mutations in our cohort affected the 5′ UTR. In mapping the 197 previously reported mutations, we find no distinct hotspots in *EYS*, although mutations clustered within or near functional domains and the putative coiled-coil. Likewise, in our cohort, the 21 distinct mutations, of which eight are novel, were all within or near functional domains.

Others have reported trends of mutations occurring more frequently in the residues closest to the C-terminus, and this region contains some of the most conserved domains of the protein, supporting the importance of the C-terminal region in normal function [3,55,56]. In analyzing the 197 previously reported mutations and the eight novel mutations reported in this study, we determined that there was only a slight bias towards mutations in the C-terminus of the protein. We found that 44% (91/205) of all mutations occurred prior to amino acid residue 1500, which encompasses the N-terminal EGF-functional domain clusters, and 56% (114/205) of mutations occurred after residue 1500, encompassing the putative coiled-coil and the C-terminal repeating Laminin G and EGF domains. Specifically, truncating mutations and splice-site mutations were most uniformly distributed of the mutation types, with 54% (7/13) of splice site mutations and 46% (46/100) of truncating mutations occurring in the first half of the protein. Missense mutations were markedly more abundant in the second half of the protein, with 34% (26/76) occurring prior to residue 1500, and 66% (50/76) occurring after. The single missense mutation reported in our cohort also occurred in the second half of the protein. Contrary to missense mutations, the majority of gross deletions occurred in the first half of the protein, which was also true of all of the deletions observed in our cohort. In total, 75% (12/16) of gross deletions involved exons originating in the first half of the protein. The significance of this was unclear, since many deletions also result in premature stop codons and truncations.

The function of EYS in humans remains largely unresolved; in *Drosophila*, the homolog SPAM, is important in development of retinal architecture, and it is postulated that this function may be conserved in humans [57]. Human EYS has been shown to play a role in stabilizing ciliary axonemes in rods and cones and maintenance of photoreceptor cells, and is expressed in four isoforms in the retina, of which two isoforms produce a protein containing all functional domains, and two produce proteins containing the N-terminal signal peptide and cleavage sites and five epidermal growth factor (EGF)-like domains [58]. The roles of each isoform in disease and how mutations across *EYS* affect the expression or function of each isoform are unknown; a better understanding of the function of *EYS* and each isoform may help explain the distribution of mutations across *EYS*.

Of the previously reported and novel mutations described in this study, the least abundant were splice-site mutations, which accounted for 6% (13/205). Splice-site mutations were overrepresented in our cohort, where they accounted for 19% (4/21) of mutations. Gross deletions, midsize deletions that typically affect one or more exons, were found to account for 8% (16/205) of mutations. Like splice-site mutations, deletions, comprising 14% (3/21) of mutations, were also overrepresented in our cohort. Truncating mutations are the most abundant mutation type observed and comprised 49% (100/205) of all mutations and 62% (13/21) of mutation types in this study. In patients with truncating *EYS* mutations, nonsense-mediated decay of mutant transcripts that results in the absence of EYS protein has been postulated to be a primary mechanism of disease; however, in some cases a truncated protein may be translated into a product with reduced or no functionality. The relative uniformity of truncating mutations across the protein supports the mechanism of nonsense-mediated decay. The second most abundant mutation type is missense mutations, which account for 37% (76/205) of all mutations. Missense mutations were underrepresented in this study’s cohort, with only one missense mutation identified involving a single patient (P14). This patient was homozygous for a recurrent mutation c.6416G>A (p.(Cys2139Tyr)) that results in the formation of a hydrogen bond and the disappearance of a twist in the tertiary structure of the protein [59]. Pathogenicity of missense mutations is difficult to establish since tolerability depends on the location and the specific change. Typically, missense mutations occur as compound heterozygotes with a truncating mutation or other non-missense mutation.

It has been hypothesized that missense mutations are generally more likely to be tolerated than truncating mutations, and as such, patients with missense mutations should present with a milder phenotype than those with more-damaging truncating phenotypes. Contradictory findings yield inconclusive results as to the validity of this hypothesis. In a study of 12 patients harboring *EYS* mutations, a correlation between severity of phenotype and the mutation type could not be established [5]. On the other hand, in 26 Japanese *EYS*-RP patients, those who had 2 truncating mutations (either homozygous for the same truncating mutations or compound heterozygous of 2 different truncating mutations) showed a more dramatic decline in visual acuity and visual field than those who carried only 1 copy of a truncating mutation [60]. In the current study, the majority of the 15 patients were heterozygous or compound heterozygotes with 2 truncating mutations, and thus the cohort was not amenable to investigate this hypothesis. The variability of severity of different missense mutations, the lack of hotspots in *EYS* and the variability of mutation types observed make genotype-phenotype relationships difficult to predict. Further study with larger cohorts and more variability in mutation types may yield greater insight into genotype-phenotype correlation.

## Figures and Tables

**Table 1:** Molecular Genetic Results for *EYS*-retinitis pigmentosa (RP) Patients.

Patient	Allele 1			Allele 2		
	Nucleotide Change	Amino Acid Change or Predicted Effect	First Reports of Mutation	Nucleotide Change	Amino Acid Change or Predicted Effect	First Reports of Mutation
P1	c.1155T>A	p.(Cys385Ter)	This Study	c.8648_8655delCATGCAGA	p.(Thr2883LysfsTer4)	[26]
P2	c.2137+1G>A	p.?	This Study	c.2137+1G>A	p.?	This Study
P3	c.490C>T	p.(Arg164Ter)	[27]	c.2260-1437_2847-6134del	p.(Ser754IlefsTer4)	This Study
P4	c.4829_4832delCATT	p.(Ser1610PhefsTer7)	This Study	c.5928-2A>G	p.?	[28]
P5 ^a^	c.7919G>A	p.(Trp2640Ter)	[1]	c.8411_8412insTT	p.(Thr2805Ter)	This Study
P6	c.32dupT	p.(Met12AspfsTer14)	[29]	c.32dupT	p.(Met12AspfsTer14)	[29]
P7	c.9286_9295delGTAAATATCG	p.(Val3096LysfsTer28)	[30]	c.2259+10539_2993-12013del	p.(Ser754AlafsTer6)	[1] ^b^
P8	c.490C>T	p.(Arg164Ter)	[27]	c.2826_2827delAT	p.(Val944GlyfsTer9)	[31]
P9	c.2889T>A	p.(Cys963Ter)	This Study	c.2889T>A	p.(Cys963Ter)	This Study
P10	c.(1766+1_17671)_(2023+1_2024-1)del	p.(Cys590TyrfsTer4)	[1] ^b^	c.(1766+1_17671)_(2023+1_2024-1)del	p.(Cys590TyrfsTer4)	[1] ^b^
P11 ^a^	c.2259+1G>A	p.?	[8]	c.8338_8342delins ^c^	p.(Gly2780_Ser2781 delinsTyrLysLeuTer)	This Study
P12	c.6528C>A	p.(Tyr2176Ter)	This Study	c.6528C>A	p.(Tyr2176Ter)	This Study
P13	c.8408dupA	p.(Asn2803LysfsTer9)	[29]	c.5834delA	p.(Lys1945SerfsTer42)	[2]
P14	c.6416G>A	p.(Cys2139Tyr)	[32]	c.6416G>A	p.(Cys2139Tyr)	[32]
P15	c.9286_9295delGTAAATATCG	p.(Val3096LysfsTer28)	[30]	c.9286_9295delGTAAATATCG	p.(Val3096LysfsTer28)	[30]

^a^ segregation analysis was performed, and mutations were confirmed to be on separate alleles; ^b^ similar to mutation previously reported by this reference; ^c^ inserted sequence: TATAAACTATAACTATAAACTATAAACTATAAACTATAAACTATAAACTATAAACTATA.

**Table 2:** Clinical Characteristics of *EYS*-RP Patients.

Patient/Gender	Ethnicity	Age at First and Most Recent Exam	Follow-Up Duration (Years) ^a^	Best Corrected Visual Acuity		Refractive Error ^b^	
				RE	LE	RE	LE
P1/F	Russian/Ukrainian	12	1	20/20	20/20	−3.25	−4.00
		13		20/20	20/20	−3.50	−3.75
P2/F	Polish/Russian	15	10	20/20	20/20	+0.25	+0.25
		25		20/20	20/20	+0.25	+0.25
P3/F	Iranian/Irish, German	18	15	20/25	20/25	−2.25	−3.00
		33		20/80	20/500	−3.75	−3.75
P4/M	Hispanic	19	27	20/20	20/20	−0.50	plano
		46		20/50	20/50	plano	+0.50
P5/F	Italian	24	15	20/30	20/25	−4.25	−4.50
		39		20/100	20/100	−2.75	−4.00
P6/M	East Indian/Iranian	24	1	20/20	20/20	n/a	n/a
		25		20/20	20/20	n/a	n/a
P7/F	Polish/Russian	32	14	20/50	20/70	n/a	n/a
		46		20/50	20/80	+2.75	+3.75
P8/M	Iranian	33	-	20/30	20/50	n/a	n/a
P9/F	Irish	34	14	20/20	20/25	−2.75	−2.50
		48		20/70	20/100	−1.25	−1.50
P10/M	Middle Eastern ^c^	36	3	20/30	20/50	n/a	n/a
		39		20/50	20/50	n/a	n/a
P11/M	Indian ^d^	38	-	20/50	20/60	−7.00	−4.00
P12/M	Italian	40	6	20/30	20/20	n/a	n/a
		46		20/25	20/25	−1.50	−2.00
P13/F	African American	48	-	20/32	20/25	−6.50	−5.75
P14/M	Chinese	47	-	HM	HM	n/a	n/a
P15/M	Ashkenazi Jewish	51	3	20/30	20/30	−1.50	−0.50
		54		20/40	20/30	−1.00	−1.25

n/a, not available; RE: right eye; LE: left eye; HM: hand motions; ^a^ years between first exam and most recent exam; ^b^ spherical equivalent; ^c^ patient has affected maternal uncles and aunt; ^d^ patient has affected sister.

## Supplementary Materials

The following are available online at www.mdpi.com/2073-4425/8/7/178/s1. Figure S1: Kinetic visual fields in *EYS* patients, Figure S2: Schematic of EYS protein showing mutational landscape.
