# Supplementary material for: EYS Mutations Causing Autosomal Recessive Retinitis Pigmentosa: Changes of Retinal Structure and Function with Disease Progression
Source: Genes (Basel). 2017 Jul 12;8(7):178. doi: 10.3390/genes8070178 (PMC5541311; doi:10.3390/genes8070178)
Supplement: Supplementary file 1 [file genes-08-00178-s001.pdf]

SUPPLEMENTARY MATERIAL

EYS Mutations Causing Autosomal Recessive Retinitis Pigmentosa: Changes of Retinal Structure and Function with Disease Progression

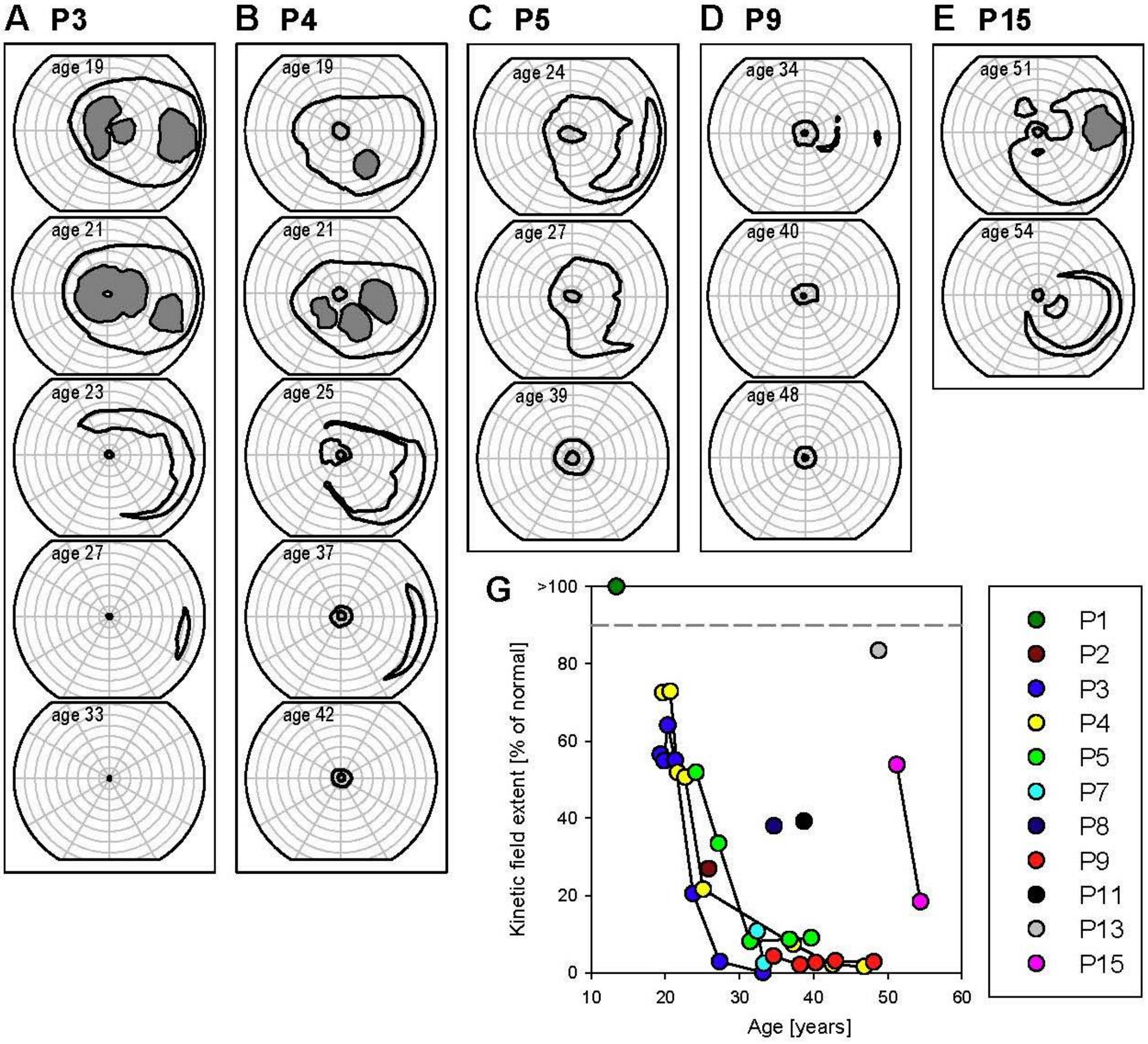

Figure S1. Kinetic visual fields in *EYS* patients. (**A-E**) Visual fields to the V-4e target and I-4e (when detectable) are depicted for a subset of patients with serial data. Scotomas to the V-4e stimulus are shaded in grey. All panels are shown as equivalent right eye visual fields. (**G**) Visual field extent of the 11 patients to the V-4e stimulus is quantified as a percentage of normal and plotted against age at visit (key with colored circles identifying patients, right). Serial data are connected by solid lines. The dashed horizontal line marks the lower limit of normal (90%).

SUPPLEMENTARY MATERIAL

EYS Mutations Causing Autosomal Recessive Retinitis Pigmentosa: Changes of Retinal Structure and Function with Disease Progression

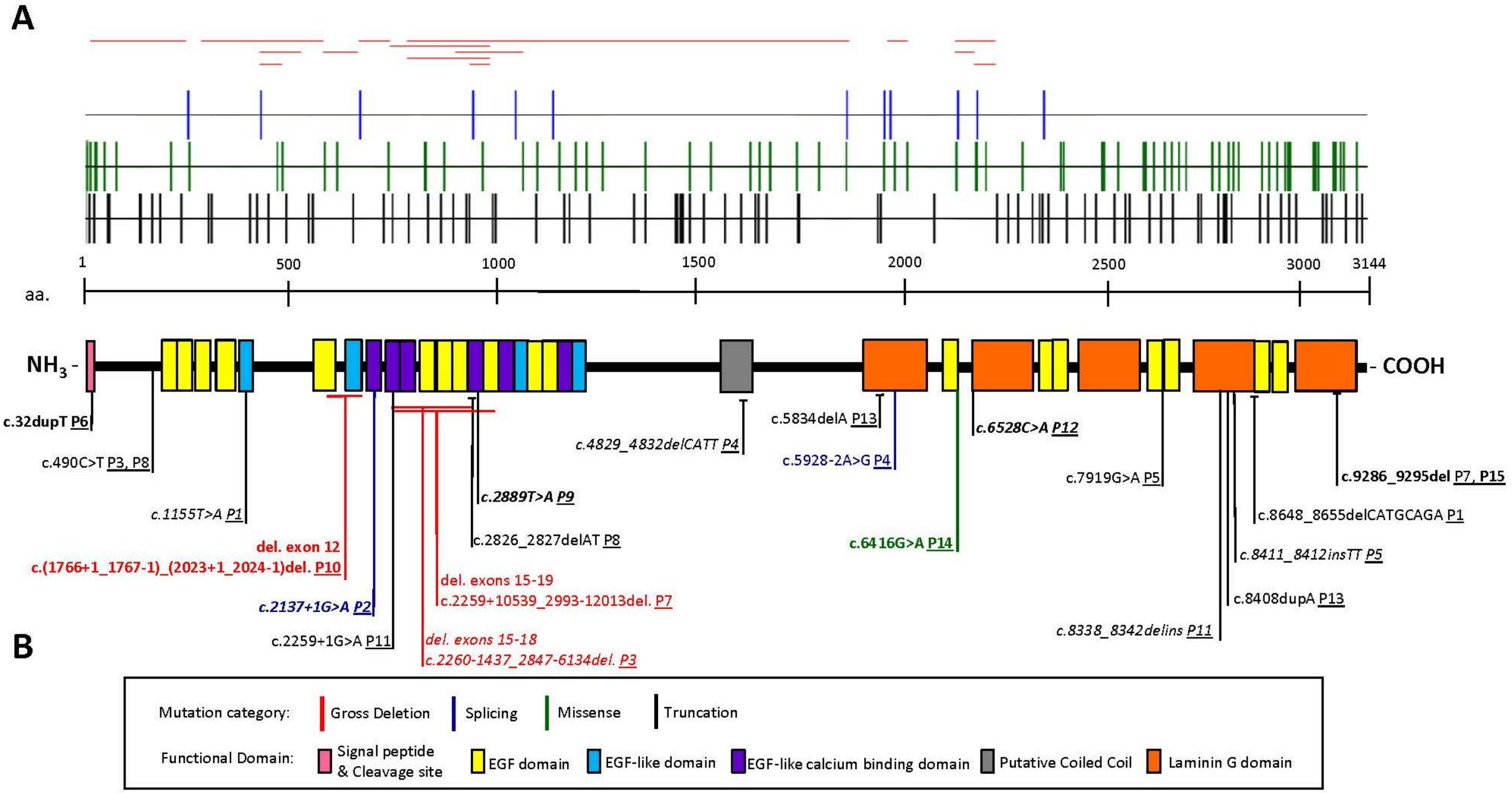

Figure S2. Schematic of EYS protein showing mutational landscape. (**A**) The approximate distribution of 197 previously reported mutations are plotted along the protein. The mutations appear on separate tracks, and are color-coded by mutation category, where, red, dark blue, dark green and black correspond to gross deletions, splice-site, missense and truncating mutations respectively. (**B**) Mutations observed in this study's cohort are mapped to the protein. The color of each mutation signifies the mutation category to which it belongs. Homozygous mutations are indicated in bold, novel mutations are italicized, and underlined patient numbers indicate the patients in this study that are associated with each mutation.
